# Interaction of Poly(l-lysine)/Polysaccharide Complex Nanoparticles with Human Vascular Endothelial Cells

**DOI:** 10.3390/nano8060358

**Published:** 2018-05-22

**Authors:** Dominik Weber, Bernhard Torger, Karsten Richter, Michelle Nessling, Frank Momburg, Beatrice Woltmann, Martin Müller, Reinhard Schwartz-Albiez

**Affiliations:** 1 Deutsches Krebsforschungszentrum (DKFZ), Clinical Cooperation Unit Applied Tumor Immunology, D-69120 Heidelberg, Germany; weberdominik@hotmail.de (D.W.); f.momburg@dkfz-heidelberg.de (F.M.); r.s-albiez@dkfz-heidelberg.de (R.S.-A.); 2 Institute of Plant and Wood Chemistry, Technische Universität Dresden, D-01737 Tharandt, Germany; bernhard.torger@tu-dresden.de; 3 Deutsches Krebsforschungszentrum (DKFZ), Central Unit Electron Microscopy, D-69120 Heidelberg, Germany; k.richter@dkfz.de (K.R.); m.nessling@dkfz-heidelberg.de (M.N.); 4 Institute of Physiological Chemistry, Faculty of Medicine Carl Gustav Carus, Technische Universität Dresden, 01307 Dresden, Germany; beatrice.woltmann@mailbox.tu-dresden.de; 5 Department Polyelectrolytes and Dispersions, Leibniz-Institut für Polymerforschung Dresden e.V., Hohe Straße 6, D-01069 Dresden, Germany; 6 Department of Chemistry and Food Chemistry, Technische Universität Dresden, D-01062 Dresden, Germany

**Keywords:** poly(l-lysine), cellulose sulfate, dextran sulfate, heparin, polyelectrolyte complex nanoparticle (PECNP), vascular endothelial cells, cell attachment, cellular uptake, proliferation

## Abstract

**Short Title:**

Polyelectrolyte nanoparticles and vascular endothelial cells.

**Abstract:**

Angiogenesis plays an important role in both soft and hard tissue regeneration, which can be modulated by therapeutic drugs. If nanoparticles (NP) are used as vectors for drug delivery, they have to encounter endothelial cells (EC) lining the vascular lumen, if applied intravenously. Herein the interaction of unloaded polyelectrolyte complex nanoparticles (PECNP) composed of cationic poly(l-lysine) (PLL) and various anionic polysaccharides with human vascular endothelial cells (HUVEC) was analyzed. In particular PECNP were tested for their cell adhesive properties, their cellular uptake and intracellular localization considering composition and net charge. PECNP may form a platform for both cell coating and drug delivery. PECNP, composed of PLL in combination with the polysaccharides dextran sulfate (DS), cellulose sulfate (CS) or heparin (HEP), either unlabeled or labeled with fluorescein isothiocyanate (FITC) and either with positive or negative net charge were prepared. PECNP were applied to human umbilical cord vein endothelial cells (HUVEC) in both, the volume phase and immobilized phase at model substrates like tissue culture dishes. The attachment of PECNP to the cell surface, their intracellular uptake, and effects on cell proliferation and growth behavior were determined. Immobilized PECNP reduced attachment of HUVEC, most prominently the systems PLL/HEP and PLL/DS. A small percentage of immobilized PECNP was taken up by cells during adhesion. PECNP in the volume phase showed no effect of the net charge sign and only minor effects of the composition on the binding and uptake of PECNP at HUVEC. PECNP were stored in endosomal vesicles in a cumulative manner without apparent further processing. During mitosis, internalized PECNP were almost equally distributed among the dividing cells. Both, in the volume phase and immobilized at the surface, PECNP composed of PLL/HEP and PLL/DS clearly reduced cell proliferation of HUVEC, however without an apparent cytotoxic effect, while PLL/CS composition showed minor impairment. PECNP have an anti-adhesive effect on HUVEC and are taken up by endothelial cells which may negatively influence the proliferation rate of HUVEC. The negative effects were less obvious with the composition PLL/CS. Since uptake and binding for PLL/HEP was more efficient than for PLL/DS, PECNP of PLL/HEP may be used to deliver growth factors to endothelial cells during vascularization of bone reconstitution material, whereas those of PLL/CS may have an advantage for substituting biomimetic bone scaffold material.

## 1. Introduction

Angiogenesis plays an important role in both soft and hard tissue regeneration, which can be modulated by therapeutic drugs. Dispersed nanoparticles (NP) can be used to deliver drugs, growth factors or genes to distinct tissues, or NP immobilized at given implants or bone substitution materials (BSM) can be used to increase their biophysical stability, biocompatibility, and cell adherence [1]. When incorporated into scaffold matrices they may additionally support their mechanical stability and enhance biocompatibility of the implanted scaffold material [2]. For both modes of application, vascular endothelial cells represent the prime cellular target as they are port of entry for drugs from the blood circulation into tissue and are essential for the vascularization of tissue reconstitution material [3]. When used as soluble carriers for e.g., anti-cancer drugs, nanoparticles coated with distinct protein ligands for specific cell surface receptors can be directed to the respective tumor tissue thereby improving the pharmacokinetics and pharmacodynamics of the respective cytotoxic drug [4,5]. In particular, encapsulation of these drugs in nanoparticles may increase their half-life in the blood circulation and enhance their concentration in the tumor tissue [6,7]. In order to reach tumor cells, nanoparticles have to overwhelm barriers of the tumor microenvironment which are represented, as one of the first steps, by the endothelial layer of blood vessels during the extravasation of the drug delivery system.

A possible strategy to enhance migration and settlement of vascular endothelial cells, thereby initiating neo-angiogenesis, could be to integrate modified nanoparticles into scaffolds. For instance, attempts have been described to stabilize and controllably release the endothelial chemokine VEGF (vascular endothelial growth factor) by binding to polysaccharide-conjugated polyelectrolyte complexes [8].

In order to find an effective nanoparticle vehicle for delivery of growth factors, cytotoxic drugsor adhesion proteins to vascular endothelial cells, several factors such as composition, size, and charge of nanoparticles with regard to their endothelial binding, uptake, and possible cytotoxicity have to be analyzed [9,10]. Various nanoparticle systems composed of charged polysaccharides, polypeptides or synthetic polyelectrolytes have been used as drug delivery systems [11,12,13] and may also support cellular ingrowth into a scaffold material [14,15,16].

Nanoparticles containing biopolymers such as polysaccharides can support controlled release of drugs and cellular adhesion within scaffold material [17,18]. Heparin based nanoparticles have been shown to improve biocompatibility of nanoparticles in various applications [19].

In our study, we applied polyelectrolyte complex nanoparticles (PECNP), composed of the polycation poly(l-lysine) (PLL) combined with the three anionic polysaccharides dextran sulfate (DS), cellulose sulfate (CS) or heparin (HEP), to human umbilical cord vein endothelial cells in vitro (HUVEC) and investigated their effect on cell binding, uptake, intracellular localization, proliferation, as well as on cellular attachment to surfaces and subsequent migration. We analyzed the influence of the composition, namely the effect of the anionic polysaccharides CS, DS, HEP within PECNP, both in the volume phase and immobilized at a model substrate. As an additional feature we used PECNP with either positive or negative net charge, which can be achieved by mixing polycation and polyanion solutions in defined ratios [11]. The potential of these PECNP with regard to directed sizing (20–500 nm), shaping (spheres, rods), and controlled charge of their surfaces has been outlined previously [11].

Adhesive coatings of PECNP were previously investigated for their loading and retarded release of distinct drugs such as bisphosphonates [12,20] and for the effect of their composition on the viability of human mesenchymal stromal cells (hMSC) [21] in the context of bone healing and remodeling. To go further, their interaction to human vascular endothelial cells (HUVEC) is now addressed in the context of angiogenesis during bone healing.

## 2. Materials and Methods

### 2.1. Preparation of Polyelectrolyte Complex Nanoparticles (PECNP)

Polyelectrolyte complex nanoparticles (PECNP) composed of the polycation poly(l-lysine) (PLL, M_W_ = 50,000 g/mol, (Sigma-Aldrich, Schnelldorf, Germany), in combination with the polyanions cellulose sulfate (CS, degree of substitution d_S_ = 2.8, M_W_ = 1,200,000 g/mol, Janssen, Beerse, Belgium), dextran sulfate (DS, d_S_ = 3.0, M_W_ = 500,000 g/mol, Carl Roth GmbH, Germany) or heparin (HEP, M_W_ = 20,000 g/mol, Carl Roth GmbH, Karlsruhe, Germany) and the fluorescein isothiocyanate (FITC) labeled PECNP species were prepared as described in detail elsewhere [11,21,22]. Briefly summarized, 2 mM clear solutions of cationic PLL and anionic CS, DS or HEP were mixed at pH = 7.0 and more or less turbid (milky) dispersions resulted. According to their molar number of charged repeating units (n+: molar number of cationic repeating units; n−: molar number of anionic repeating units) two molar mixing ratios denoted n−/n+ were applied, which were n−/n+ = 0.9 and n−/n+ = 1.1. These two molar mixing ratios resulted in PECNP, which had a slightly positive net charge (PECNP-0.9, n−/n+ = 0.9, cationic excess) or slightly negative net charge (PECNP-1.1, n−/n+ = 1.1, anionic excess), which was proven by zeta-potential measurements (see below). Furthermore, sizes i.e., hydrodynamic radii R_H_ of these PECNP were determined by dynamic light scattering (see below). The particle sizes of the herein used PECNP can be found in the Table 1.

### 2.2. Dynamic Light Scattering (DLS)

Hydrodynamic radii (R_H_) of PECNP were determined by DLS using Jianke Portable Particle Sizer (Jianke Instruments Co. Ltd., Wuhu, China). A scattering angle of 89.3° was applied. Two mL of PECNP dispersions were filled in cuvettes with a circular bottom (D = 10 mm). The autocorrelation function was recorded for 180 s and R_H_ values were calculated from values of the translational diffusion coefficient D using the Stokes–Einstein equation R_H_ = k_B_T/(D 6 πη) (Boltzmann constant k_B_; temperature T = 298 K; and viscosity η). The ALV-5000/E/EPP-Software (ALV GmbH, Langen, Germany) was used for the computation of DLS parameters applying the “Simple Fit” tool. Intensity weighted size distributions were computed and plotted. Error values of R_H_ given in Table 1 were related to the standard deviation of (3–14) individual but equally prepared PECNP samples.

### 2.3. Zeta-Potential

The Zeta-potential measurements on PECNP dispersions in order to determine the net charge of PECNP were performed at neutral pH values (pH = 7.4) using a Zetasizer 3000 (Malvern Instruments Ltd., Worchestershire, UK).

### 2.4. Cells

HUVEC (human umbilical cord vein endothelial cells, PromoCell, Heidelberg, Germany) were cultivated in endothelial cell basal media full medium (ECBM-FM; PromoCell) consisting of endothelial cell basal media supplemented with 10% fetal bovine serum (FBS) and endothelial cell growth medium supplement (PromoCell). Cells were cultured at 37 °C, 5% CO_2_ atmosphere and 95% relative humidity. HUVEC were split before reaching confluence and were used up to cell culture passage 6 for functional assays. HUVEC were detached using the Detach Kit (PromoCell) according to the manufacturers’ protocol. In detail, cells were washed with HEPES (4-(2-hydroxyethyl)-1-piperazineethanesulfonic acid) and subsequently with trypsin/EDTA (Ethylenediaminetetraacetic acid) for a few seconds. Cells were then detached by applying fresh trypsin/EDTA to the cells. As soon as cells started to detach the cell culture flask was gently tapped to detach the remaining adherent cells. The enzymatic reaction was stopped using trypsin neutralizing solution (PromoCell). All cells were cultured under sterile conditions at 37 °C, 5% CO_2_ atmosphere, and 95% relative humidity. Fresh media exchanges were performed twice a week.

### 2.5. Growth Pattern of HUVEC on PECNP

PECNP (100 µL/cm^2^ of a 2 mM PECNP dispersion) were immobilized on the bottom of an 8-well-µ-slide, ibiTreat (Ibidi, Martinsried, Germany) on a waving platform shaker under sterile conditions overnight at room temperature. HUVEC (4 × 10^4^ cells in 300 µL basal ECBM medium) were seeded into the wells and photographic microscopy images were acquired after 0, 10, and 20 h of incubation using a ZEISS cell observer. (Carl Zeiss, Oberkochen, Germany). 

### 2.6. Cellular Uptake of Immobilized PECNP (Light Microscopy)

Fluorescein isothiocyanate (FITC)-labeled PECNP (52.6 µL/cm^2^ of a 2 mM dispersion) were immobilized under motion on a 24-well plate overnight. Then, wells were washed with phosphate buffered saline (PBS), 4 × 10^4^ HUVEC were seeded on the PECNP layer in 1 mL basal ECBM and incubated at 37 °C and 5% CO_2_ and 95% relative humidity for 24 h. The media were then replaced with fresh basal ECBM and cells were incubated for another 24 h. For further analysis, cells were detached and transferred to an 8-well-µ-slide–ibiTreat. After 24 h of incubation in ECBM-FM, cells were fixed as indicated below.

For fluorescence microscopic analysis of the cellular uptake of dispersed PECNP, 2.3 × 10^4^ cells were seeded on an 8-well µ-slide–ibiTreat and FITC-labeled PECNP were added to the attached cells for 1 h, 4 h, and 24 h after exchange of culture media to ECBM. The uptake of PECNP was stopped by washing the cells with Ca^2+^/Mg^2+^-containing PBS followed by fixation with 4% paraformaldehyde (PFA) in PBS (*v/v*) for 10 min at room temperature. After 3 washing steps in PBS, 4 drops of iTFx Signal Enhancer (Thermo Fisher, Dreieich, Germany) were applied for 30 min to block unspecific binding. HUVEC were then incubated with biotinylated anti-CD31 mouse mAb (PECAM-1, platelet/endothelial cell adhesion molecule 1; Becton&Dickenson (B&D), Heidelberg, Germany) for 30 min followed by a Cy3-coupled streptavidin antibody (Dianova, Hamburg, Germany) for 30 min. Cell nuclei were stained with Hoechst 33342 (Biotium, Fremont, CA, USA) for 10 min. For preservation, fluorescent mounting media (Dako, Hamburg, Germany) was added to the cells. Cells were analyzed using a widefield microscope (Zeiss Cell Observer Z1; Carl Zeiss, Oberkochen, Germany) and a confocal microscope (Leica TCS SP5 II, Wetzlar, Germany).

### 2.7. Analysis of Cellular Interactions with PECNP (Transmission Electron Microscopy, TEM)

ACLAR film slides (Redding, CA, USA) were placed in 24-well plates and 1 × 10^5^ HUVEC were seeded on top in 1 mL media and incubated for 24 h at 37 °C, 5% CO_2_, and 95% relative humidity. A volume of 25 µL of a 2 mM PECNP dispersion was added to the media (final dilution 1:40) for 4 h. Incubation was stopped by the addition of a primary fixative (4% formaldehyde,2% glutaraldehyde and 1 mM MgCl_2_, in 100 mM sodium phosphate buffer at pH 7.2). Following two post-fixations steps, buffered OsO_4_ (1%) and ethanolic uranyl acetate (0.5%), respectively, samples were flat-embedded in epoxy resin (Serva, Heidelberg, Germany) for ultrathin sectioning. Sections were post-stained with uranyl and lead and investigated by TEM (EM912, Carl Zeiss, Oberkochen, Germany).

### 2.8. Analysis of Cellular Interactions with Fluorescent PECNP (Flow Cytometry)

Detached HUVEC were incubated with PE-conjugated mouse antibodies against CD31 on ice for 30 min in the dark. For setting parameters of live-dead discrimination, HUVECs were incubated in parallel with 4% PFA in another reaction tube. After two washing steps, cells were resuspended in 100 µL of basal culture medium (i.e., ECBM) containing FITC-labeled PECNP (1:40 dilution of a 2 mM stock suspension) and incubated for 1 h. After careful washing, cells were resuspended in a 1:15 dilution of BD-via probe^TM^ (B&D, Heidelberg, Germany) for exclusion of dead cells. Flow cytometric analysis was performed using a BD FACS Canto^TM^ II (BD Biosciences, San Jose, CA, USA), analyzing 50.000 cells/sample, and data were analyzed by the FlowJo^TM^ software (Version 10, Ashland, OR, USA). Background values for PE- and FITC-fluorescence were calculated using unstained cells. In order to analyze PECNP internalization, cells incubated with FITC-labeled PECNP were analyzed for their fluorescence signal intensity. Then, trypan blue was added to the samples and the signal intensity was re-analyzed resulting only in the excitation of the internalized PECNP as the signal of PECNP on the surface was quenched [23]. For analysis of uptake kinetic, HUVEC were incubated with FITC-labeled PECNP for 5, 30, 60, or 120 min. HUVECs were first analyzed for the total amount of cell-associated FITC-label (PECNP signal). Then, 120 µL of a 0.4 mg/mL solution of trypan blue was added to the cells and samples were immediately measured again. Unlabeled HUVEC were used as negative control (threshold value for FITC-signal). Each measurement was performed for 10,000 cells.

### 2.9. Analysis of Cell Proliferation ([^3^H]-Thymidine Incorporation Assay)

PECNP in several concentrations (2.5, 5, 10, 50 µL/cm^2^ of a 2 mM PECNP stock dispersion, diluted in sterile water) were immobilized on a 96-well tissue culture plate. For this, PECNP were applied to the bottom of the wells of a tissue culture plate under sterile conditions, and incubated overnight under motion on a waving platform shaker at room temperature. HUVEC were seeded in triplicates in media without heparin (Promocell culture media supplement DM2, PromoCell, Heidelberg, Germany) on PECNP-coated 96-well plates at a cell density of 3.5 × 10^3^ cells/well. Cells were allowed to adhere for 2–4 h and then 1 µCi [^3^H]-thymidine/well was applied. For analyzing the influence of dispersed PECNP on proliferation, HUVEC were seeded into 96-well plates, incubated at 37 °C and 5% CO_2_ and 95% relative humidity for 1 h and 10 µL of PECNP solution in the concentrations as indicated above was added to the culture media. Then, 1 µCi [^3^H]-thymidine was added to the media. All tissue culture plates were incubated under cell culture condition for 24–26 h. The incubation was stopped as cells were detached and frozen. For this, the cell media was carefully removed and 200 µL of a solution of trypsin-EDTA (Detach kit, PromoCell) was applied to the well. The plates were incubated at 37 °C for 5–10 min and the cells were subsequently frozen at −20 °C. An additional thawing–freezing step was applied and the cell debris (including the cellular DNA) was transferred to a printed filter mat A (PerkinElmer, Waltham, MA, USA) using a TomTec harvester (TomTec, Hamden, CT, USA). The dried filters were transferred to a sample bag (PerkinElmer) and the [^3^H]-thymidine incorporated into DNA was measured using a Betaplate scintillator and a MicroBeta TriLux scintillator counter (both PerkinElmer).

## 3. Results and Discussion

We analyzed the influence of composition and net charge of biorelated polyelectrolyte complex nanoparticles (PECNP) on human umbilical cord vein endothelial cells (HUVEC) with regard to their effect on cell adhesion, uptake, and cytotoxicity. Biorelated cationic poly(l-lysine) (PLL) was combined with the three biorelated anionic polysaccharides cellulose sulfate (CS), dextran sulfate (DS), and heparin (HEP) resulting in the PECNP of PLL/CS, PLL/DS, and PLL/HEP. The interaction to HUVEC was studied for PECNP in the volume phase as well as bound at model substrates such as tissue culture plates. In the following, first colloidal properties of the used PECNP systems are introduced (Section 3.1), second, results on the interfacial interaction between HUVEC and immobilized PECNP coatings (Section 3.2), and third interaction between HUVEC and PECNP in the volume phase (Section 3.3) are shown. Finally, proliferation of HUVEC in contact to PECNP in both states will be screened (Section 3.4).

### 3.1. Colloidal Properties of PECNP

Six PECNP systems composed of PLL/CS, PLL/DS, and PLL/HEP with positive and negative net charge, indicated as PLL/polysaccharide-0.9 (n−/n+ = 0.9) or PLL/polysaccharide-1.1 (n−/n+ = 1.1), respectively, were prepared. Their colloidal parameters hydrodynamic radius (R_H_), from dynamic light scattering (DLS) measurements, as well as net charge from zeta-potential measurements are summarized in Table 1.

Generally, the R_H_ values (sizes) of all PECNP ranged between 62–106 nm, where PECNP of PLL/CS (101–106 nm) were considerably larger, and those of PLL/DS (69–88 nm) and PLL/HEP (62–67 nm) smaller. However, given the obvious molecular weight (M_W_) differences of the polyanions (see Materials and Methods section) no correlation between M_W_ (CS > DS > HEP) and PECNP size (CS > DS > HEP) should be drawn within the given error range. Hence for all compositions HUVEC should encounter PECNP with diameters D_H_ = 2 R_H_ between ≈ 120–210 nm. Expectedly, PECNP with slight excess of polycation (n−/n+ = 0.9, PEC-0.9) featured positive, while those with slight excess of polyanion (n−/n+ = 1.1, PEC-1.1) featured anionic net charge at pH = 7.4, which was detected by zeta-potential measurements. The extended zeta-potential courses versus pH are given in the Supplementary Material (SM, Figure SM1a–d) for PLL/CS and PLL/DS (data on PLL/HEP not shown). Obviously, for all compositions the cationic PECNP (n−/n+ = 0.9) showed a drop of positive zeta-potential beginning at around pH = 9, which is due to the deprotonation of PLL primary amino groups having isoelectrical point IEP ≈ 9.5. Whereas, anionic PECNP (n−/n+ = 1.1) kept their negative zeta-potential for the whole pH range at values in the range −50 to −40 mV.

Furthermore, the conformation state of PLL within PECNP was studied both in the volume phase by circular dichroism (CD) spectroscopy and bound to polystyrene (PS) films relevant for the cell culture experiments by attenuated total reflection Fourier transform infrared (ATR-FTIR) spectroscopy. In Figure SM2 CD spectra on 0.002 M dispersions of PLL/CS-1.1, PLL/DS-1.1 and PLL/HEP-1.1 are given, which all show a doublet at 208/225 nm with negative intensity as well as a positive signal at 195 nm, both diagnostic for the α-helical secondary structure (similar data for PLL/CS-0.9, PLL/DS-0.9, and PLL/HEP-0.9 is not shown). Additionally, in the Figure SM3 ATR-FTIR spectra on respective PECNP samples bound to PS films show Amide I and Amide II maxima at 1653 and 1546 cm^−1^, which are also diagnostic for the α-helical secondary structure of PLL (similar data for PLL/DS-0.9 and PLL/HEP-0.9 is not shown). The PLL conformation state for all six PECNP samples is included in the Table 1.

Finally, scanning force microscopy (SFM) images of spin-coated PECNP at poly(styrene) (PS) films after rinsing in HEPES buffer given in the Figure SM4 of the SM show dense coverage and granular morphology of merged and single PECNP, which is summarized in the Table 1. Interestingly, for PLL/CS-0.9 (Figure SM4a) the presence of both microparticles and nanoparticles can be identified, where the microparticle seems to be composed of nanoparticles (“blackberry model”) as was also claimed for complexes between synthetic polyelectrolytes earlier [24].

Conclusively, all three PECNP compositions PLL/CS, PLL/DS, and PLL/HEP for both positive (n−/n+ = 0.9) and negative (n−/n+ = 1.1) net charge did not show substantial differences in their colloidal behavior with respect to size and net charge magnitude and also on the secondary structure as well as morphology level there were no significant differences. Hence in the following in-vitro experiments on the PECNP interaction with HUVEC, argumentation on a chemical level should be justified.

### 3.2. Cell Growth on Immobilized PECNP Coatings

**Figure 1:**
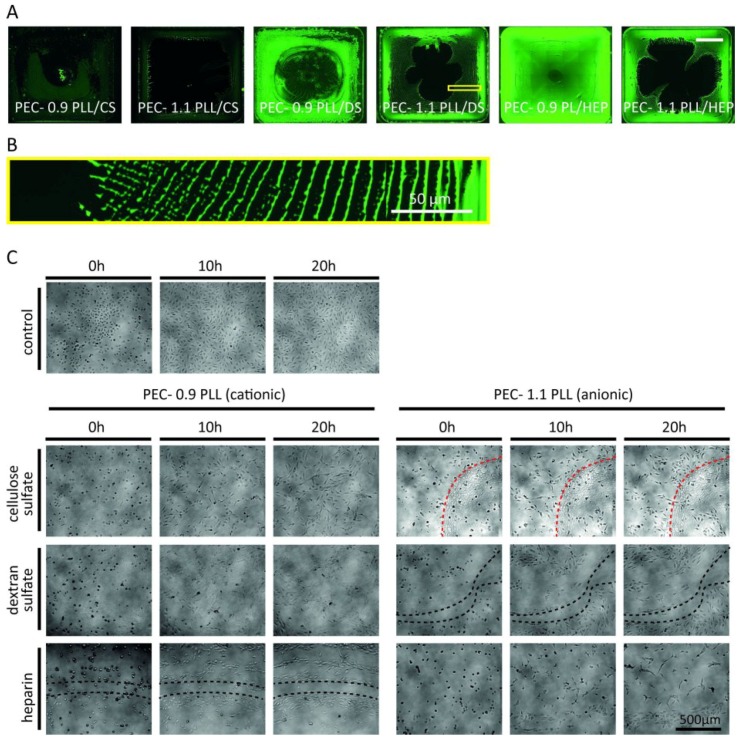
Influence of immobilized polyelectrolyte complex nanoparticles (PECNP) on human umbilical cord vein endothelial cells (HUVEC) seeding. Microscopic images of PECNP distribution on various areas of slide surface (scale bar: 250 μm). Cationic (0.9) or anionic (1.1) PECNP composed of poly(l-lysine) (PLL) and either cellulose sulfate (CS), dextran sulfate (DS) or heparin (HEP) were used. (**A**) PECNP in some cases attached to the surface during the drying process in ring-shaped patterns which entailed areas of high and low PECNP concentration (Scale bar: 250 µm); (**B**) Magnification of PEC-1.1 PLL/DS from A—ring-shaped pattern of dried PECNP; (**C**) PECNP immobilized on Ibidi-slides at high concentrations (100 μL/cm^2^ of a 2 mM PECNP dispersion). PECNP distributed inhomogeneously on the surface of the 8-well µ-slide–ibiTreat slides during the drying process. HUVEC cells (4 × 10^4^ cells) were seeded onto the slides and photos were taken at times indicated. Depending on the modification and concentration of PECNP applied, alterations in cell growth and adhesion patterns were observed. Cells avoided areas with higher concentrations of PECNP as outlined by black dashed lines. This effect was most prominent for PECNP composed of PLL/DS-1.1 and of PLL/HEP-0.9. Cells grew in a looser, web-shaped pattern on PECNP of PLL/CS. High concentrations of PLL/CS (red dashed line) had less effect than PLL/DS or PLL/HEP PECNP.

In order to see what impact these PECNP in immobilized state have on the growth behavior of HUVECs , surfaces of various cell culture dishes and microscopic slides were coated with PECNP, modified by CS, DS, and HEP. Additionally, for fluorescence microscopy and flow cytometric analysis also PECNP labeled with FITC were applied. Light microscopy of immobilized PECNP on slides revealed, that even under constant motion during coating, PECNP did not spread and attach homogeneously on the bottom of 8-well ibiTreat slides, coated with 100 μL/cm^2^ of a 0.4 mM dispersion of the respective PECNP, which can be obtained from Figure 1A and 1B. HUVEC seemed to avoid areas coated with high concentrations of PECNP. This was also demonstrated by coating the slides with a higher concentration of PECNP (100 μL/cm^2^ of a 2 mM dispersion). Cell attachment in areas with high concentration of PECNP (ring-shaped areas) was less dense as compared to those with lower concentration of PECNP, which is shown in kinetics of cell growth on these slides (Figure 1C). This effect was most prominent for PECNP composed of PLL/HEP and PLL/DS (Figure 1C, black dashed line), which grew in a web-shaped manner. As compared to control cultures without PECNP-coating, cells grew in a looser, reticular pattern on PLL/CS PECNP (Figure 1C, dashed red line).

### 3.3. Cell Growth on Dispersed PECNP in the Volume Phase

#### 3.3.1. Cell Surface Attachment of PECNP

**Figure 2:**
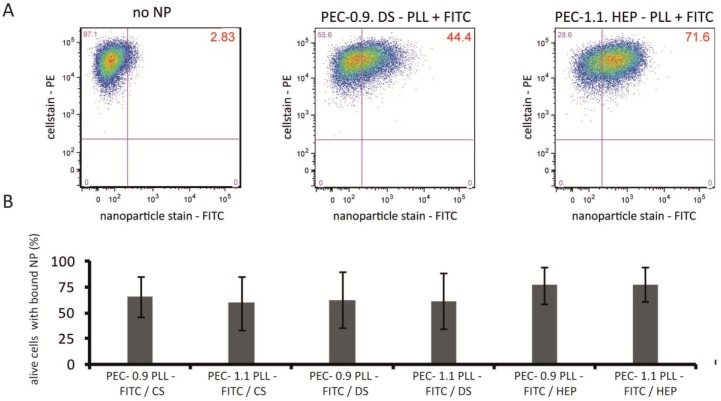
Interaction of polyelectrolyte complex nanoparticles (PECNP) with the cell surface of human umbilical cord vein endothelial cells (HUVEC). HUVEC were incubated on ice for 1 h with fluorescein isothiocyanate (FITC)-labeled PECNP, cells were stained with an anti-CD31 monoclonal antibody conjugated to PE and subjected to flow cytometric analysis. (**A**) Cells were live-gated by VIA-probe and gated according to CD31-PE surface staining (*y*-axis) and FITC staining (*x*-axis). As negative control, cells without FITC-labeled PECNP were taken and gates set accordingly. Cells with bound FITC-labeled PECNP were counted in comparison to negative control (percentage of positive cells given for each analysis, right upper quadrant); (**B**) Percentage of cells with surface-bound PECNP compared to control without PECNP set as 100%, mean ± SD, number of experiments: *n* = 4. CS = cellulose sulfate, DS = dextran sulfate, HEP = heparin-containing PECNP.

The next issues to be studied were whether and to what extent PECNP in the volume phase attach to the cell surface and are further taken up by HUVECs. The attachment process was quantified by flow cytometric analysis using fluorescein isothiocyanate (FITC)-labeled PECNP in dispersion. For measuring cell surface attachment, HUVEC were incubated with FITC-labeled PECNP on ice for 1 h and the amount of PECNP on the cell surface of live cells was estimated (Figure 2). Apparently, cell surface attachment of PECNP of PLL/HEP, regardless of net charge was slightly better than that of PLL/CS and PLL/DS. An obvious reason for this may be that heparin is an endogenous molecule of mammalian physiology and that heparin may interfere with surface receptors on HUVEC like VEGF-R2 or the hyaluronic acid receptor for endocytosis (HARE) [25]. Thus the charge of interfering particles with, as a rule, slightly negatively charged cell surfaces is less decisive than functional interaction with a specific cell surface receptor for binding efficacy.

#### 3.3.2. Cellular Uptake of PECNP

**Figure 3:**
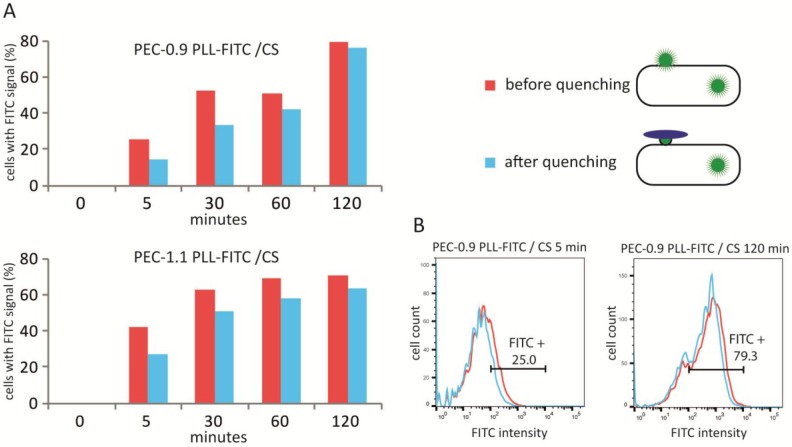
Analysis of polyelectrolyte complex nanoparticles (PECNP) internalization into human umbilical cord vein endothelial cells (HUVEC). FITC-labeled PECNP were incubated with HUVEC for 5 to 120 min, HUVEC were detached and immediately prepared for flow cytometric analysis. Cells were live-gated (VIA-probe) and further gated according to their scatter signals (forward (FSC) vs. sideward scatter signal (SSC)). Cells without PECNP incubation were taken as controls to set the background threshold for PECNP-FITC interaction. Cells were measured and the percentage of cells with bound PECNP in relation to the controls was determined (red bars in (**A**) histograms, red lines in (**B**) flow cytometric diagrams). By the addition of trypan blue, the cell surface attached FITC signal was quenched and flow cytometric analysis was repeated, measuring only the FITC signal within the cells (blue bars in (**A**), blue lines in (**B**) and as exemplified in the upper right scheme).

In a next step, the uptake of PECNP was measured in comparison to cell surface attachment in a kinetic experiment (Figure 3). In this experiment, PECNP composed of PLL/CS were picked out and incubated for up to 120 min with HUVECs under cell culture conditions. The cell surface attachment was distinguished from uptake by quenching the FITC signal on the cell surface by trypan blue.

**Figure 4:**
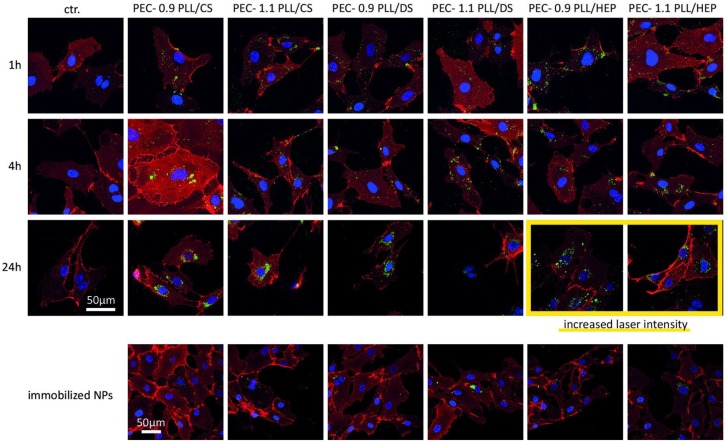
Uptake of polyelectrolyte complex nanoparticles (PECNP) into human umbilical cord vein endothelial cells (HUVEC). HUVEC were investigated for their uptake of PECNP-FITC (fluorescein isothiocyanate), either from the volume phase or from the immobilized state, demonstrated by confocal microscopy. Cells were cultivated either with PECNP in endothelial medium (volume phase) for times indicated or in contact with immobilized PECNP (surface) for 48 h cultivation time (bottom row). After cultivation, cells were detached and transferred to Ibidi slides and stained with Hoechst 33342 (cell nuclei) and an anti-CD31 (PECAM-1) monoclonal antibody conjugated to biotin, followed by incubation with an anti-mouse antibody conjugated to Cy3/streptavidin.

Uptake of PECNP for all three combinations PLL/CS, PLL/DS and PLL/HEP was also observed in confocal microscopy using both PECNP in the volume phase and immobilized on a model substrate (Figure 4). In a time range of 1 h to 24 h it became apparent, that for all compositions and net charges PECNP were taken up by HUVEC. However, the composition influenced the uptake rate of soluble PECNP. PECNP of PLL/HEP were taken up very early and fast, leading to big clusters already after 1 h incubation. This result was in accordance with our observations shown in Figure 2, where PLL/HEP attached on live cells already after 1 h of incubation. Remarkably, after 24 h of incubation the fluorescence signal of internalized PECNP of PLL/HEP faded in contrast to that of PLL/DS and PLL/DS, which may be due to degradation of the PLL/HEP PECNP by endogenous heparinases of endothelial cells [26] while PLL/CS and PLL/DS PECNP may be non-digestible by endothelial cells. After 24 h incubation, the PECNP signal strength, regardless of net charge and composition, was concentrated to the largest extent in the perinuclear area of the cells. The PECNP were exclusively situated in this endo-lysosomal compartment but none in the cytosol or in the nucleus which was reported for charged polystyrene based nanoparticles [27]. Also, surface-immobilized PECNP were taken up by the cells though to a much smaller extent than of soluble PECNP and within a longer time frame (Figure 4).

**Figure 5:**
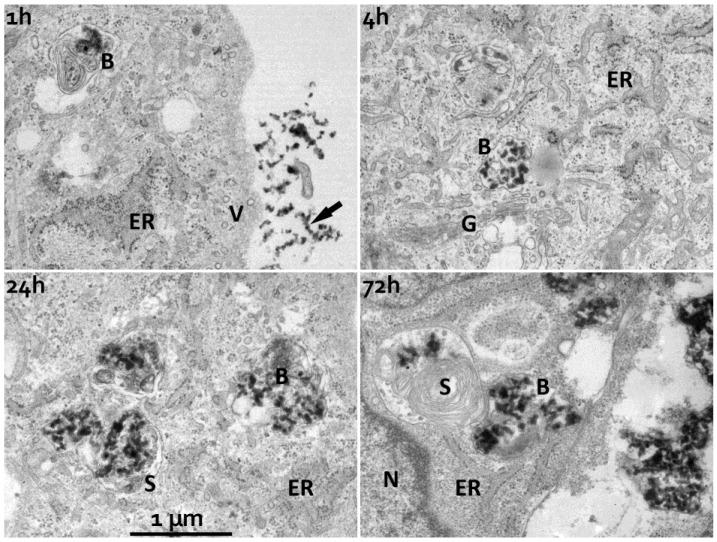
Ultrastructural demonstration of the polyelectrolyte complex nanoparticle PECNP compartment in human umbilical cord vein endothelial cells (HUVEC). HUVEC cell cultures admixed with PECNP composed of positively charged poly(l-lysine)/cellulose sulfate (PLL/CS-0.9) in the volume phase were fixed for TEM measurements after incubation times as indicated (1 h/4 h/24 h/72 h). 1 h/4 h incubation (upper row): disperse clusters of bead-like PECNP are in contact with the cells. Individual beads are compact, of variable shape and of typically 40 nm size. They frequently connect to each other into short chains (arrow). Close contact with the cell surface does not involve particular cell-surface coats. Internalized beads (B), i.e., PECNP, are rarely seen after 1 h incubation, more readily within the 4 h-samples, and always within membrane-enclosed vesicles. These vesicles differ in size from the typical endothelial vesicles of HUVEC. 24 h/72 h incubation (bottom row): vesicles with internalized PECNP grew beyond 1 µm-size. This “nanobead compartment” now occupied an essential volume of the cell cytoplasm. The PECNP-vesicles also feature inclusions of stacked membranes (S) which appear segregated from the bead-clusters. These membrane convolutions get more prominent with increasing time. B: (nano)beads/PECNP; ER: rough endoplasmatic reticulum; N: cell nucleus, G: Golgi apparatus V: endosomal vesicles, all four images at same magnification.

In more detail, transmission electron microscopy (TEM) showed neat attachment of PECNP to the plasma membrane of cells, preferentially in clustered form, and internalized PECNP in vesicles. PECNP in plasma membrane transit had not been seen unambiguously. In particular, none of the many coated vesicles abundant in HUVEC cell periphery ever contained PECNP indicating that internalization is not receptor-mediated in contrast to previously published data on charged nanoparticles which were taken up via the clathrin-mediated pathway [28]. This observation may also be in contrast to another report which describes that differently composed polyelectrolyte nanoparticles have an affinity to caveolae during endothelial cell uptake [29]. No apparent cell surface damage by binding and uptake of PECNP was observed by TEM. The observed vesicles increased with time of incubation, indicating that newly internalized PECNP-containing vesicles fuse with older ones. After long-time incubation, e.g., 72 h, huge vesicles occupied a great portion of the cytoplasmic space. Stuffed with PECNP, they also included stacked membranes, while both components appeared segregated within the same membrane-bound space (Figure 5).

The observation that PECNP accumulated with time in large endosomal vesicles indicates that lysosomal digest did not efficiently take place. Distinct endothelial proteins, such as PECAM, integrated into vesicles may be crucial for further intracellular processing [30]. Thus, it may be that the PLL/polysaccharide composition of PECNP does not suffice for further intracellular processing of nanoparticles, at least in endothelial cells, but additionally requires specific cell-specific proteins integrated into the PECNP as mentioned above. Also, it has to be considered that incubation with PECNP for several days, which may not occur under physiological conditions, represents an extreme situation and was performed to clearly demonstrate the intracellular localization and showed that lysosomal degradation did not take place. As has been shown by light microscopy and flow cytometry, uptake happened in shorter times (5–120 min).

**Figure 6:**
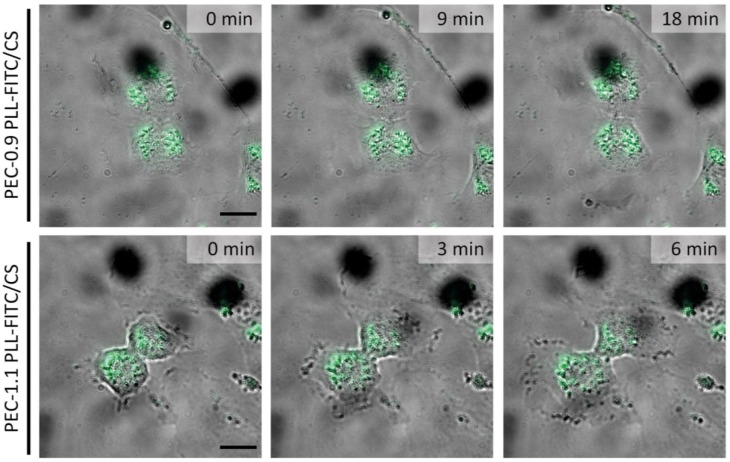
Distribution of internalized polyelectrolyte complex nanoparticles (PECNP) during cell division. Human umbilical cord vein endothelial cells (HUVEC) were incubated with 300 μL each of 50 μM fluorescein isothiocyanate (FITC)-labelled PECNP dispersed in endothelial culture medium for 24 h as described in Materials and Methods. After 3 washing steps, cells carrying PECNP were analyzed in the telophase of cell division by widefield microscopy. Images show separation of cells loaded with PECNP at time points indicated. The number of PECNP within cells apparently was split in approximately equal portions during cell division. PEC: polyelectrolyte complex; PEC-1.1: negatively charged PECNP; PEC-0.9: positively charged PECNP; PLL: (poly-l)lysine; CS: cellulose sulfate; (Scale bar: 20 μm).

In contrast to physical measurement of particle size (Table 1), size estimation by TEM revealed somewhat smaller sizes for single PECNP (extracellular: 39 ± 9 nm; intracellular after 4 h incubation: 50 ± 8 nm; intracellular after 24 h incubation: 42 ± 7nm). Interestingly, the incorporated FITC-labeled PECNP were equally distributed during mitosis (Figure 6).

### 3.4. Effect of PECNP on Cell Proliferation in the Volume Phase and Immobilized State

**Figure 7:**
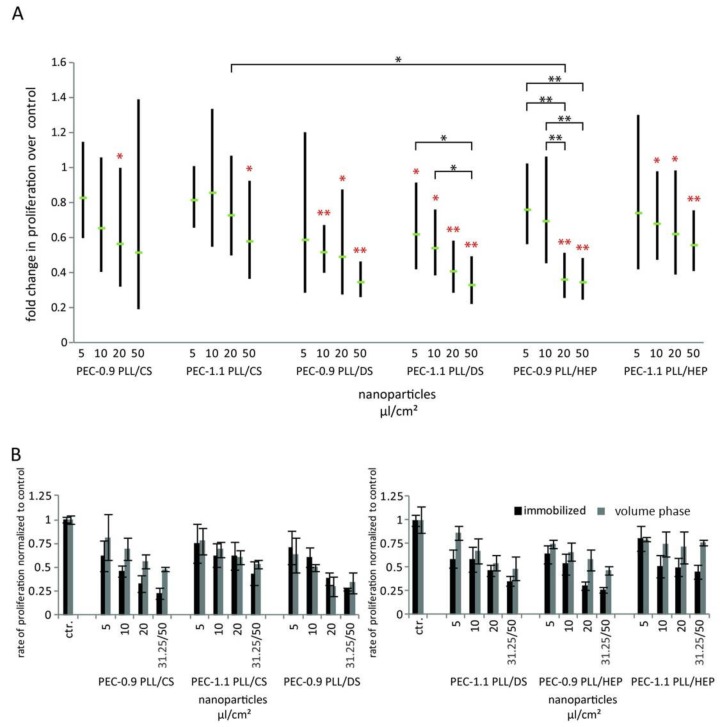
The influence of polyelectrolyte complex nanoparticles PECNP on cell proliferation. The influence of PECNP on proliferation of human umbilical cord vein endothelial cells (HUVEC) was assayed by [3H] thymidine incorporation as described in Materials and Methods. (**A**) Influence on proliferation, shown as fold change for each PECNP preparation compared to untreated (i.e., without PECNP addition) control HUVEC cultures. HUVECs were seeded on culture plates coated with PECNP. Black bars indicate 95% confidence interval (number of experiments: *n* = 4), red * indicate significant changes in proliferation using a two-tailed, paired *t*-test. Horizontal bars and black * indicate significant differences between various concentrations of the same PECNP applied or between various PECNP of same concentration (ANOVA, one way analysis of variance), * *p* < 0.05, ** *p* < 0.01) (**B**) Direct comparison of HUVEC proliferation rates affected by PECNP in the immobilized state (black bars) and in the volume phase (grey bars). Proliferation rates were normalized to those of control cultures (ctr). The highest concentration of PECNP in fluid state was 31 μL/cm^2^, and for immobilized PECNP 50 μL/cm^2^. The other concentrations of PECNP were as indicated. Error bars indicate standard deviation of at least triplicates/assay.

Since PECNP were taken up in large amounts, it was analyzed whether they may influence cell proliferation both in the volume phase and attached on a surface. To this end, the influence of PECNP on proliferation of HUVEC was tested by their [^3^H]-thymidine uptake during a cultivation time of 24 h (Figure 7). All PECNP samples reduced proliferation in a dose-dependent fashion which was evident both for the immobilized as well as for the volume phase state though the negative influence was stronger for the immobilized PECNP (black bars Figure 7B). PLL/HEP and PLL/DS PECNP had significant anti-proliferative effects at medium and high concentration (20 and 50 µL/cm^2^, respectively). Negatively charged PLL/CS PECNP (PEC-1.1), especially in lower concentrations (5 and 10 µ/cm^2^), had the lowest anti-proliferative effect. Reflected in reduced proliferation rates after PECNP uptake, the time-dependently high endosomal accumulation of PECNP may have caused stress for the cells. We did not find a significant difference with regard to uptake and intracellular localization between negatively and positively charged nanoparticles as has been reported [26]. However, as estimated by analysis of TEM images it appeared, that positively charged PECNP showed a larger number of vesicles with particles than negatively charged PECNP after 24 and 72 h incubation (data not shown), which may be due to the slightly negatively charged cell membrane. It was suggested that charged nanoparticles of various materials are more cytotoxic than uncharged ones which may however not apply for polysaccharide based nanoparticles as for instance those containing chitosan [10].

In our study, all PECNP, in the volume phase and in an immobilized state reduced cell proliferation in HUVECs in a concentration-dependent manner albeit to various extents. In another study cytotoxic effects of iron oxide nanoparticles on human endothelial cells were reported [31]. Although in our study the proliferation rate was reduced by addition of PECNP, it has to be stressed that this did not entail a higher cytotoxicity. Flow cytometric analysis of dead versus live cells after application of PECNP did not show a higher propensity for dead cells (data not shown). Also, the fact that mitosis was not influenced by internalized PECNP supports this finding. As the intracellular load of PECNP is apparently bisected after each cell division the non-digestible PECNP will gradually be diminished. In another study uptake of modified polystyrene nanoparticles by mesenchymal stem cells did not affect the viability of these cells [13].

Immobilized PECNP can be used as an integrative part of a scaffold material and hence it is important to know how endothelial cells are able to adhere to and migrate on a PECNP coated surface. Application of PECNP to surfaces of culture dishes or microscopic slides resulted in heterogeneous spreading of nanoparticles. Under these conditions PECNP had generally an anti-adhesive effect. This repulsive effect was lowest with PECNP of PLL/CS, which suggests these nanoparticles are more appropriate for coating of scaffold surfaces. A further advantage for in-vivo application may be that both, HEP and CS have anti-coagulant activities [32,33]. In an earlier study we investigated the interaction between PECNPs and human mesenchymal stromal cells (hMSC) and found, similar to the findings of this study, that net charge did not significantly influence adhesion and uptake, while PECNP composition affected e.g., metabolic activity of hMSC [21]. It has also been shown that PECNP can be applied as drug delivery system when cast as particle films [12,20,34,35]. In our study we found differential effects of PECNP on endothelial cell adhesion. Therefore, when applying PECNP in cast form for surface mediated drug delivery the possible anti-adhesive effect of several PECNP has to be considered and certainly those with lowest anti-adhesive effect have to be preferred for this purpose.

## 4. Conclusions

Our study showed that PLL/polysaccharide based PECNP attached to the cell surface of vascular endothelial cells and were taken up into the vesicular compartment in a cumulative manner. Among the three PECNP compositions used here, PLL/HEP seemed to be the ideal vehicle for transport of drugs or growth factors to endothelial cells, because (i) attachment and uptake was faster than for the other modifications, (ii) PLL/HEP seemed to be digested in the lysosomal compartment by endogeneous glycosidases and (iii) possibly sulfatases, and (iii) interactions with growth factors, important for vascularization, such as vascular endothelial growth factor (VEGF) and basic fibroblast growth factor (bFGF), have a high binding capacity for heparin, and thus PECNP of PLL/HEP may be helpful for supporting the presentation of these growth factors [36] to vascular endothelial cells and may also reduce release of the growth factors during cellular regeneration for example of bone tissue within scaffolds [14] (iii). On the other side, PECNP of PLL/CS may be better suited for coating surfaces of scaffold material since its anti-adhesive effects and its anti-proliferative impact were much smaller compared to PECNP composed of PLL/HEP or PLL/DS. Depending on the specific requirements for nanoparticle application, mixing of HEP and CS in ternary complexes of PLL/CS/HEP in appropriate fractions may be advantageous to design specific properties for several applications as mentioned here. Finally, the net charge of the PECNP, which can be modulated by the excess of either cationic PLL or one of the three anionic polysaccharides, did not play an essential role in our experimental approach, which is in line with former results on the interaction of PECNP with comparable compositions with human mesenchymal stromal cells (hMSC) [21].

Our study has yielded basic insights into the biocompatibility of PECNP for vascular endothelial cells. In future experiments, using PECNP, the influence of shear stress and stretched conditions has to be taken into account, since it is known that under flow conditions, equivalent to the physiological condition in the blood stream [37], uptake of nanoparticles may be different [38,39]. In an earlier study, by measuring uptake of silica nanoparticles into human endothelial cells it was found, that cytotoxicity was not increased (i) and endocytosis was decreased as compared to maintenance of cells under static conditions (ii) [40]. So far, we were able to show interactions with endothelial cells in the volume phase via the comparatively harsh shear stress conditions of flow cytometry confirming data of other groups [23]. Therefore, adhesion and uptake may be lower under dynamic than under static conditions and thereby anti-proliferative effects may also be diminished in the in-vivo situation. Also, in-vivo studies have to clarify the potential clearance of PECNP from the body with time.

## Figures and Tables

**Table 1:** Hydrodynamic radius (R_H_), zeta-potential at pH = 7.4, PLL conformation and morphology (dry spin coated state at poly(styrene) (PS) film) of the PECNP samples used in this work. Numbers of individual measurements are given in brackets. The data have been partly already given in [21], whereby slight deviations within the error range might prevail.

PECNP Sample	R_H_/nm	Zeta-Potential/mV	PLL Conformation	Morphology
PLL/CS-0.9	106 ± 7 (#9)	+45 + 5 mV	α-helical	granular, spherical
PLL/DS-0.9	69 ± 12 (#14)	+45 + 5 mV	α-helical	-
PLL/HEP-0.9	62 ± 8 (#3)	---	α-helical	-
PLL/CS-1.1	101 ± 10 (#13)	−43 + 5 mV	α-helical	granular, spherical
PLL/DS-1.1	88 ± 17 (#8)	−44 + 5 mV	α-helical	granular, spherical
PLL/HEP-1.1	67 ± 7 (#6)	---	α-helical	granular, spherical

## Supplementary Materials

The following are available online at http://www.mdpi.com/2079-4991/8/6/358/s1, Figure SM1: Zeta-potential/pH profiles of PECNP, Figures SM2 and SM3: PLL conformation in PECNP samples, Figure SM4. Morphology of PECNP at poly(styrene) (PS) found by scanning force microscopy (SFM).
