# Supplementary material for: Interaction of Poly(l-lysine)/Polysaccharide Complex Nanoparticles with Human Vascular Endothelial Cells
Source: Nanomaterials (Basel). 2018 May 22;8(6):358. doi: 10.3390/nano8060358 (PMC6027445; doi:10.3390/nano8060358)
Supplement: Supplementary file 1 [file nanomaterials-08-00358-s001.pdf]

nanomaterials

Supporting Material

Interaction of poly(L-lysine)/polysaccharide complex nanoparticles with human vascular endothelial cells

Dominik Weber [1], Bernhard Torger [2], Karsten Richter [3], Michelle Nessling [3], Frank Momburg [1], Beatrice Woltmann [4], Martin Müller [5,6,*] and Reinhard Schwartz-Albiez [1]

1 Deutsches Krebsforschungszentrum (DKFZ), Clinical Cooperation Unit Applied Tumor Immunology, D-69120 Heidelberg, Germany; dominik.weber@dkfz.de (D.W.); f.momburg@dkfz-heidelberg.de (F.M.); r.s-albiez@dkfz-heidelberg.de (R.S.A.)
2 Technische Universität Dresden, Institute of Plant and Wood Chemistry , D-01737 Tharandt, Germany; bernhard.torger@tu-dresden.de;
3 Deutsches Krebsforschungszentrum (DKFZ), Central Unit Electron Microscopy , D-69120 Heidelberg, Germany; k.richter@dkfz.de (K.R.); m.nessling@dkfz.de (M.N.)
4 Technische Universität Dresden, Faculty of Medicine Carl Gustav Carus, Institute of Physiological Chemistry, 01307 Dresden, Germany; beatrice.woltmann@mailbox.tu-dresden.de (B.W.)
5 Leibniz-Institut für Polymerforschung Dresden e.V., Department Polyelectrolytes and Dispersions, ,Hohe Straße 6, D-01069 Dresden, Germany
6 Technische Universität Dresden, Department of Chemistry and Food Chemistry, D-01062 Dresden, Germany
* Correspondence: mamuller@ipfdd.de; Tel.: +49-351-4658-405; Fax: +49-351-4658-284

1. Zeta-potential/pH profiles of PECNP

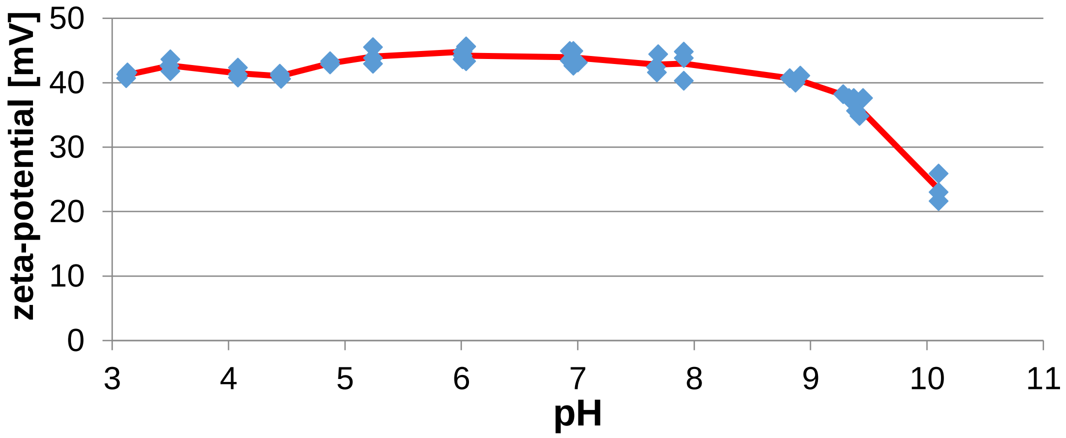

(**a**)

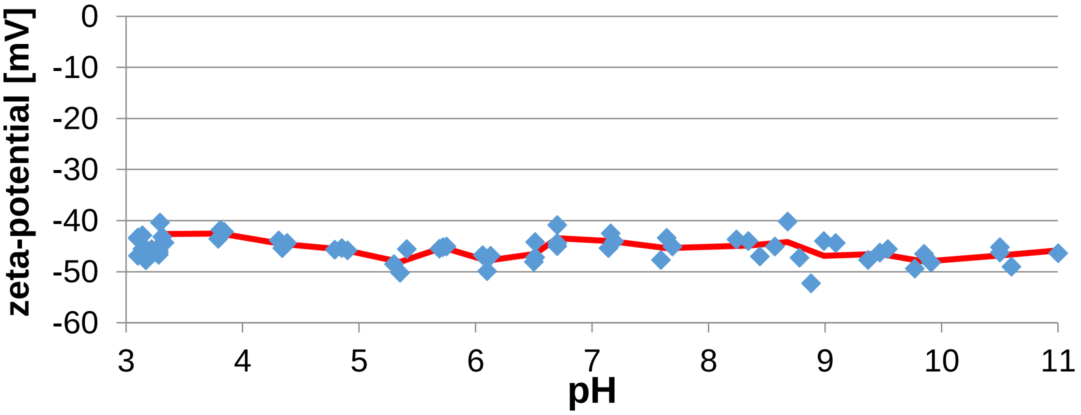

(**b**)

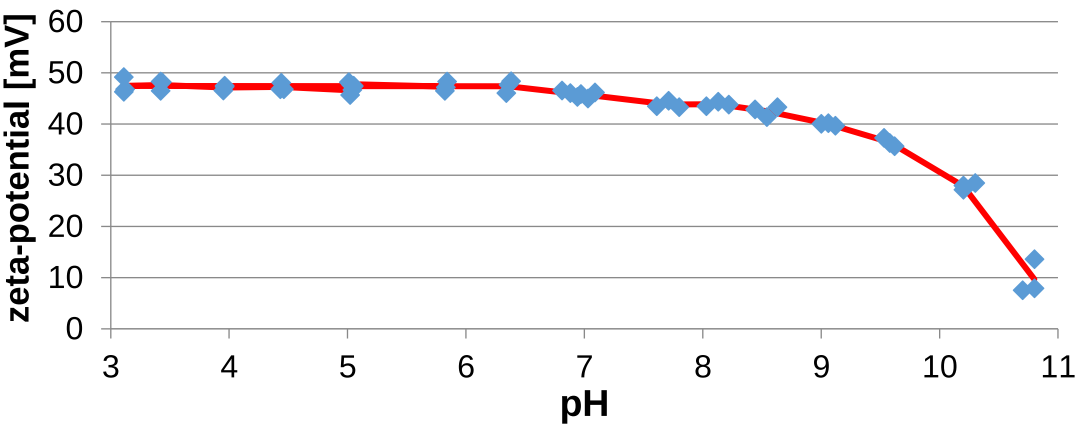

(**c**)

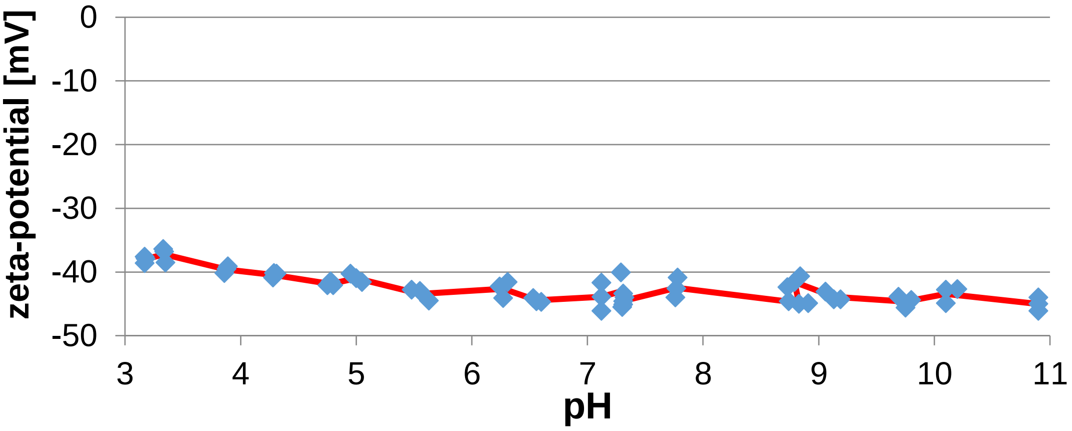

(**d**)

Figure SM1. Zeta-potential/pH profiles of PECNP: (**a**) PECNP-0.9 PLL/CS-2m, native pH = 6.95; (**b**) PECNP-1.1 PLL/CS-2mM, native pH = 6.61; (**c**) PECNP-0.9 PLL/DS-2mM, native pH = 6.10; (**d**) PECNP-1.1 PLL/DS-2mM, native pH = 7.19.

2. PLL conformation in PECNP samples

2.1. CD spectroscopy at dispersed PECNP in the volume phase

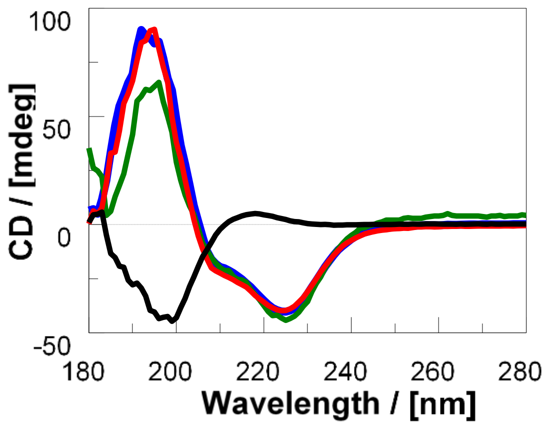

Figure SM2. CD spectra on pure PLL solution (black) and PECNP dispersions of PLL/CS (blue), PLL/DS (red) and PLL/HEP (green) for n-/n+ = 1.1, concentration c = 0.002M and pH=7.4.

2.2. ATR-FTIR spectroscopy at PECNP bound to polystyrene (PS) substrate

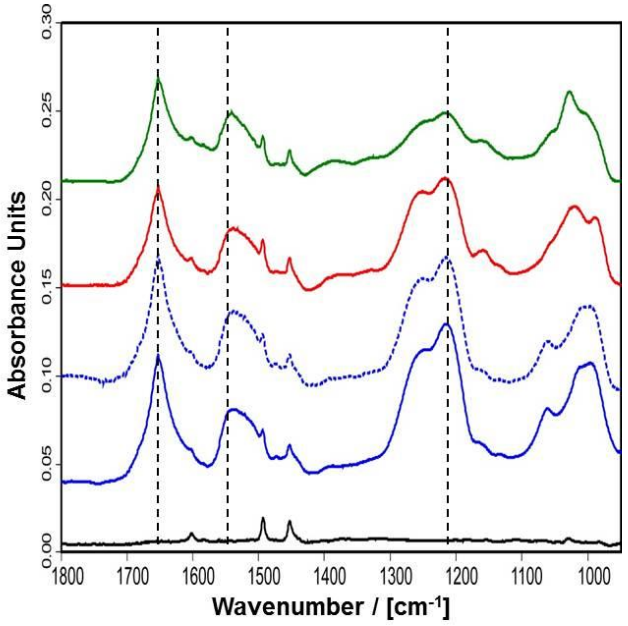

Fig. SM3. ATR-FTIR spectra on spin-coated PS film (at Ge model substrate) (black) and thereupon casted PECNP dispersions of PLL/CS-1.1 (blue, full), PLL/CS-0.9 (blue, broken), PLL/DS-1.1 (red) and PLL/HEP-1.1 (green), concentration c = 0.002M after rinsing in HEPES buffer (pH = 7.4).

3. Morphology of PECNP at poly(styrene) (PS) found by scanning force microscopy (SFM)

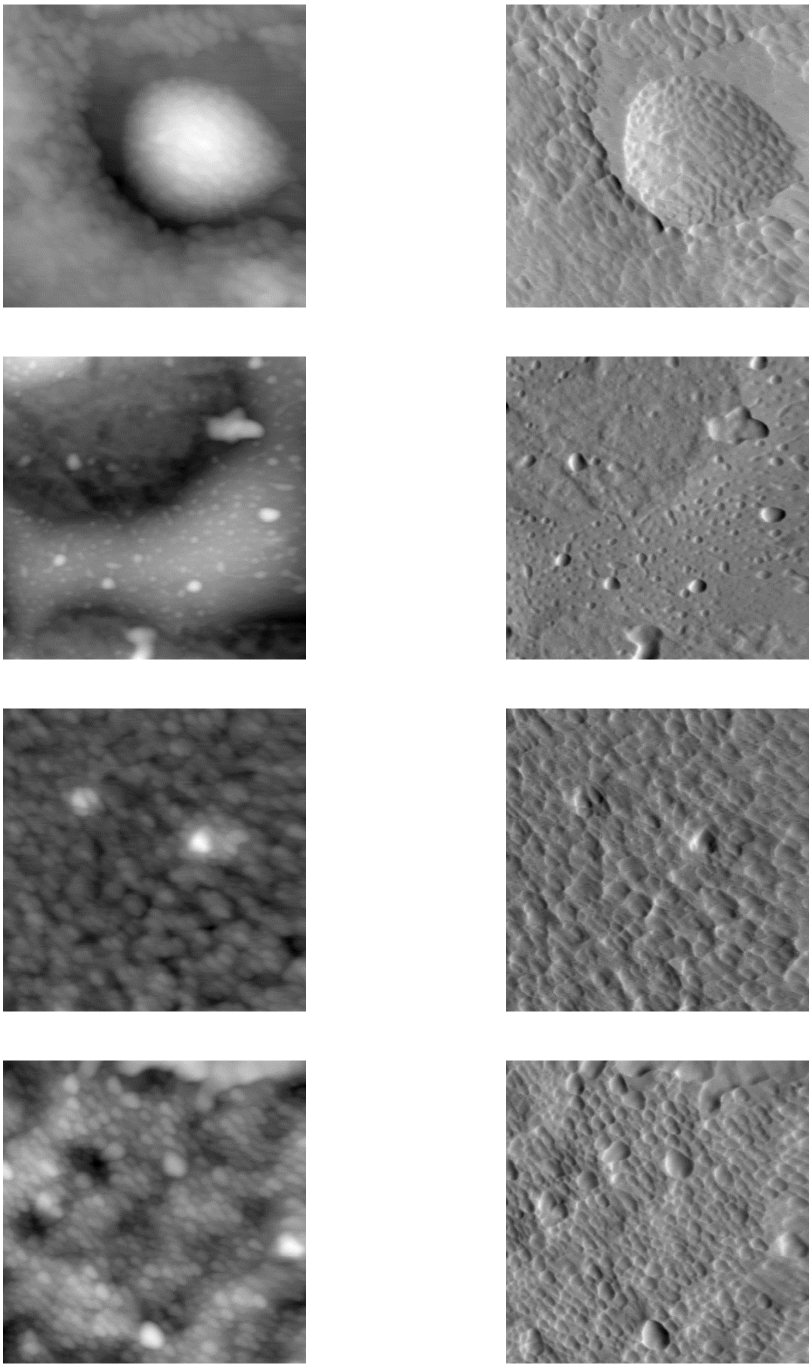

(**a**)

(**b**)

(**c**)

(**d**)

Fig. SM4. Morphology of PECNP at poly(styrene) (PS) found by scanning force microscopy (SFM): SFM images (2 x 2 ◎m; topography images top row; error images, bottom row) of casted PLL/CS-0.9 (**a**), PLL/CS-1.1 (**b**), PLL/DS-1.1 (**c**) and PLL/HEP-1.1 (b) dispersions (0.002M, 50 microliters, pH = 7.4) at polystyrene (PS) film spin-coated onto germanium model substrate after rinsing in HEPES buffer. The samples are identical to Fig. SM3.

© 2018 by the authors. Submitted for possible open access publication under the terms and conditions of the Creative Commons Attribution (CC BY) license (http://creativecommons.org/licenses/by/4.0/).
